# To Avoid Night Calls

**Published:** 1885-10

**Authors:** 


### TO AVOID NIGHT CALLS.

A good story is going the rounds (who started it we do not know,) at the expense of the young physician who is always so busy that he does not know what to do:

"I have got more business than I can attend to," boasted he to an old practitioner, who knew he lied. "I had to get out of bed five times last night." "Why don't you buy some insect powder?" quietly asked the old doctor.—*Medical Age.*

